# Selective serotonin reuptake inhibitors (SSRIs) prevent meta-iodobenzylguanidine (MIBG) uptake in platelets without affecting neuroblastoma tumor uptake

**DOI:** 10.1186/s13550-020-00662-w

**Published:** 2020-07-08

**Authors:** Thomas Blom, Rutger Meinsma, Marja Rutgers, Corine Buitenhuis, Marieke Dekken-Van den Burg, André B. P. van Kuilenburg, Godelieve A. M. Tytgat

**Affiliations:** 1 Princess Máxima Center for Pediatric Oncology, Utrecht, The Netherlands; 2 Gastroenterology & Metabolism, Department of Clinical Chemistry, Amsterdam University Medical Center, Amsterdam, The Netherlands; 3 Department of Experimental Therapy, Netherlands Cancer Institute, Amsterdam, The Netherlands; 4 Department of Pediatric Oncology, Emma Children’s Hospital, Amsterdam University Medical Center, Amsterdam, The Netherlands

**Keywords:** Meta-iodobenzylguanidine (MIBG), Hematological toxicity, Thrombocytopenia, Platelets, Selective serotonin reuptake inhibitor (SSRI)

## Abstract

**Background:**

The therapeutic use of [^131^I]meta-iodobenzylguanidine ([^131^I]MIBG) is often accompanied by hematological toxicity, mainly consisting of persistent and severe thrombocytopenia. While MIBG accumulates in neuroblastoma cells via selective uptake by the norepinephrine transporter (NET), the serotonin transporter (SERT) is responsible for cellular uptake of MIBG in platelets. In this study, we have investigated whether pharmacological intervention with selective serotonin reuptake inhibitors (SSRIs) may prevent radiotoxic MIBG uptake in platelets without affecting neuroblastoma tumor uptake.

**Methods:**

To determine the transport kinetics of SERT for [^125^I]MIBG, HEK293 cells were transfected with SERT and uptake assays were conducted. Next, a panel of seven SSRIs was tested in vitro for their inhibitory potency on the uptake of [^125^I]MIBG in isolated human platelets and in cultured neuroblastoma cells. We investigated in vivo the efficacy of the four best performing SSRIs on the accumulation of [^125^I]MIBG in nude mice bearing subcutaneous neuroblastoma xenografts. In ex vivo experiments, the diluted plasma of mice treated with SSRIs was added to isolated human platelets to assess the effect on [^125^I]MIBG uptake.

**Results:**

SERT performed as a low-affinity transporter of [^125^I]MIBG in comparison with NET (*K*_m_ = 9.7 μM and 0.49 μM, respectively). Paroxetine was the most potent uptake inhibitor of both serotonin (IC_50_ = 0.6 nM) and MIBG (IC_50_ = 0.2 nM) in platelets. Citalopram was the most selective SERT inhibitor of [^125^I]MIBG uptake, with high SERT affinity in platelets (IC_50_ = 7.8 nM) and low NET affinity in neuroblastoma cells (IC_50_ = 11.940 nM). The in vivo tested SSRIs (citalopram, fluvoxamine, sertraline, and paroxetine) had no effect on [^125^I]MIBG uptake levels in neuroblastoma xenografts. In contrast, treatment with desipramine, a NET selective inhibitor, resulted in profoundly decreased xenograft [^125^I]MIBG levels (*p* < 0.0001). In ex vivo [^125^I]MIBG uptake experiments, 100- and 34-fold diluted murine plasma of mice treated with citalopram added to isolated human platelets led to a decrease in MIBG uptake of 54–76%, respectively.

**Conclusion:**

Our study demonstrates for the first time that SSRIs selectively inhibit MIBG uptake in platelets without affecting MIBG accumulation in an in vivo neuroblastoma model. The concomitant application of citalopram during [^131^I]MIBG therapy seems a promising strategy to prevent thrombocytopenia in neuroblastoma patients.

## Background

**Fig. 1:**
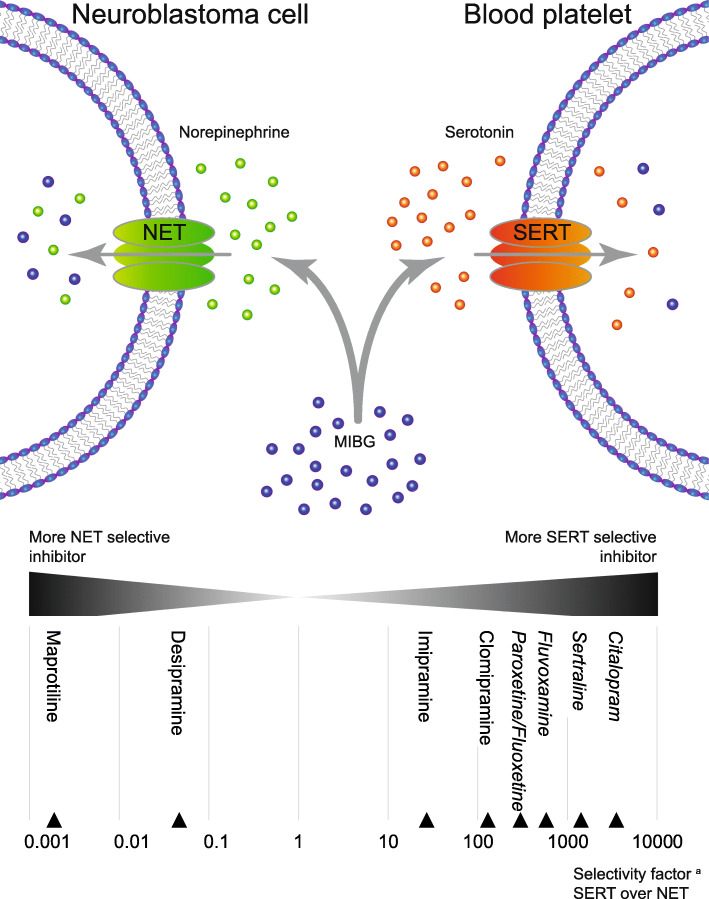
Uptake of MIBG by NET in neuroblastoma cells and SERT in platelets. Norepinephrine (in green) is the natural substrate of the norepinephrine transporter (NET), serotonin (in red) of the serotonin transporter (SERT). Both monoamine transporters are capable of intracellular transport of MIBG (in blue). ^a^For all monoamine transporter inhibitors, the selectivity factor was calculated from the ratio of the dissociation constants (*K*_D_) for serotonin at SERT and norepinephrine at NET [4]. Selective serotonin reuptake inhibitors (SSRIs) are depicted in italics

Neuroblastoma, originating from the early developing embryonic sympathetic nervous system, is the most common extracranial solid tumor of childhood [1]. Despite intensive multimodality treatment, the overall survival rate for high-risk neuroblastoma patients remains poor [2]. Approximately 90% of neuroblastomas express the norepinephrine transporter (NET) [3], enabling the application of meta-iodobenzylguanidine (MIBG), a structural analog of norepinephrine, for imaging and treatment purposes (Fig. 1). Whereas the iodine-123 radiolabeled form is used as a highly selective radioactive imaging agent [5], radioactive iodine-131 MIBG is being used since 1984 in patients with relapsed or refractory neuroblastoma to achieve a tumor response [6]. This treatment in these patients has been accompanied by hematological toxicity, mainly consisting of persistent and severe thrombocytopenia [7–9].

Numerous studies have shown a clear association between the whole-body radiation dose from [^131^I]MIBG treatment and the severity of hematological toxicity [8–10]. However, in a recent study consisting of 213 high-risk neuroblastoma patients treated at a single institution, no such relation was observed [11]. Furthermore, thrombocytopenia after [^125^I]MIBG treatment, a radiopharmaceutical with negligible radiation exposure to nontargeted neighboring cells [12], was unforeseen and far more severe than expected from calculated whole-body radiation doses in neuroblastoma patients [13, 14]. It appears that it is not the iodine-125 in itself that leads to hematological toxicity but the combination of the radioiodine with MIBG, since in patients with advanced colon cancer, high doses of iodine-125-labeled monoclonal antibody did not cause bone marrow toxicity, while the iodine-131-labeled antibody caused severe hematological toxicity in 26% of patients [15, 16].

It is conceivable that radiation exposure after selective uptake of MIBG by platelet precursor cells, the bone marrow megakaryocytes, is the major cause of MIBG treatment-associated thrombocytopenia. Human platelets possess the serotonin transporter (SERT, see also Fig. 1) [17], and [^131^I]MIBG accumulates in human platelets as effectively as the natural substrate serotonin [18]. The aim of this study was to investigate whether prevention of radiolabeled MIBG accumulation in platelets is feasible in vivo, without reducing tumor uptake in nude mice bearing neuroblastoma xenografts.

## Methods

### Overexpression of human SERT and NET in HEK293 cells

NET (addgene plasmid #15475) and SERT (addgene plasmid #15483) were transiently expressed in HEK293 cells (ATCC® CRL-3216™, Manassas, USA) using X-tremeGENE™ HP DNA Transfection Reagent (Sigma-Aldrich, St. Louis, USA). To investigate the expression of NET and SERT in the transfected cells, SDS/PAGE was performed on 4–12% polyacrylamide gel (Lonza, Basel, Switzerland) followed by Western blotting on 0.45 μm nitrocellulose (Amersham, Buckinghamshire, UK). Antibody incubation was performed for 1 h with primary antibodies against NET (NET 17-1) and SERT (ST51-1; both from MAb Technologies, Stone Mountain, USA) and with IRDye® 800CW Donkey anti-Mouse IgG (Li-cor) as secondary antibody. The Odyssey Imaging System (Li-cor, Lincoln, USA) was used for immunoblot imaging.

### Platelet isolation

Blood was obtained from patients with polycythemia vera or secondary polycythemia. All volunteers gave their informed consent. Platelet-rich plasma (PRP) was prepared as described previously [18]. Results of uptake and inhibition studies were compared to results obtained with PRP prepared from the blood of healthy volunteers as control.

### [^3^H]serotonin and [^125^I]MIBG uptake experiments

Uptake studies of [^3^H]serotonin and [^125^I]MIBG in transfected HEK293 cells were performed on cells cultured in 6 wells (9.5 cm^2^ each) plates. Two days after plating, at a confluence of 80–90%, the culture medium was removed and fresh medium containing MIBG (range 0.05–20 μM) or serotonin (range 0.5–50 μM) was added with a fixed concentration of radioactivity of 3.7 kBq/ml. Cells were co-incubated with monoamine transporter inhibitors at final concentrations ranging from 10^−10^–10^−4^ M. Nonspecific uptake was determined in HEK293 cells transfected with an empty vector alone. After 1 h of incubation at 37 °C, the cells were washed twice with Hank’s Balanced Salt Solution (HBSS), after which the cell-associated radioactivity was extracted with 0.2 M sodium hydroxide and counted in Ultima Gold® (PerkinElmer, Waltham, USA) scintillation fluid with a PerkinElmer Tri-Carb liquid scintillation counter (4910TR).

Uptake studies of [^3^H]serotonin and [^125^I]MIBG in platelets were performed as previously described [18]. Vials of 0.5 ml PRP contained on average 0.97 ± 0.12 × 10^8^ platelets (mean ± SD, *n* = 17) to which 3.7 kBq/ml of radiolabeled serotonin or MIBG was added, supplemented with cold MIBG to a final concentration of 10^−8^ M. The different monoamine transporter inhibitors were added at final concentrations ranging from 10^−12^ to 10^−4^ M. Platelets were incubated at 37 °C for 15 min (serotonin) or 4 h (MIBG), after which the platelets were spun down and washed and radioactivity was counted as described above.

The human neuroblastoma cell line SK-N-SH (ATCC® HTB-11™) and the rat pheochromocytoma cell line PC12 (ATCC® CRL-1721™), both expressing NET [19], were routinely cultured in 6-well culture plates [20]. The highly differentiated neuroadrenergic PC12 cells were included to investigate the role of cytoplasmic storage granules. [^125^I]MIBG uptake and inhibition experiments were performed in PC12 cells, which are rich in storage granules and do, in this respect, resemble platelets, and in SK-N-SH cells, which contain few storage granules [20]. All experiments were conducted as previously described [18].

Total uptake was calculated as a percentage of the added radioactivity and expressed relatively to the uptake of cells without inhibitor. Nonspecific uptake of substrate was determined by co-incubating cells with excess imipramine (30 or 4 μM imipramine for platelets and neuroblastoma, respectively) [18].

### Effect of the monoamine transporter uptake inhibitors on the [^125^I]MIBG tumor uptake in vivo

Female athymic BALB/c nu/nu mice were bred in the animal facility of the Netherlands Cancer Institute. Experiments were performed in accordance with the national regulations for animal experimentations and approved by the local animal welfare committee. Subcutaneous (s.c.) neuroblastoma tumors consisted of either first passages of intrasplenic-induced SK-N-SH xenografts or later passages from s.c. propagation of the xenograft [19]. The model of SK-N-SH neuroblastoma-xenografted mice has been shown to be clinically relevant due to its selective MIBG tumor retention and sensitivity to therapeutic [^131^I]MIBG dosages [19, 21]. The tumor volume doubling time was on average 5 days. The toxicity of each monoamine transporter inhibitor was assessed in 2 to 5 non-tumor-bearing nude mice by 1 h careful observation following intraperitoneal (IP) injection of the monoamine transporter inhibitor. Applied inhibitor doses were based on earlier studies (summarized in Electronic Supplementary Material: Table I) and varied from 2 to 50 mg/kg. Provided that no toxicity was observed, plasma of these animals was subsequently analyzed in the ex vivo bioassay described below. The effect of the monoamine transporter inhibitors on the MIBG biodistribution was studied in xenografted mice of 10–14 weeks old (mean body weight 24.0 g), and the average tumor size was to 0.23 g (range 0.14–0.30 g). First, mice were treated IP with either a monoamine transporter inhibitor or sodium chloride (control). Thirty minutes later, they received an injection in the tail vein with 1 μg MIBG spiked with 4.0–8.0 kBq [^125^I]MIBG. One hour after administration of the radiopharmaceutical, the animals were bled from the carotid artery under ether anesthesia and dissected. Tumors and tissues of interest were blotted to remove adhering blood, weighted, and gamma counted. The [^125^I]MIBG tissue levels were expressed as the percentage of the injected radioactivity dose per gram tissue. Mice bearing PC12 pheochromocytoma xenografts induced by direct s.c. injections of PC12 cells were used in similar uptake and inhibition experiments.

### Ex vivo bioassay of murine plasma SSRI activity

The biological activity of the SSRI in the circulation of the nude mice was analyzed using a bioassay similar to that described for humans [22, 23] which determines the inhibitory potency of the murine plasma (containing the SSRI) on the [^125^I]MIBG uptake in human platelets. One hour after the SSRI administration, mice blood was centrifuged to obtain the plasma, which was stored at − 20 °C until analysis. For each SSRI, plasma of 2 to 5 animals was pooled. In the bioassay, we mixed 500 μl human PRP with 5 μl or 15 μl murine plasma to obtain 34- to 100-fold dilutions of the murine plasmas. Pooled plasma from 6 untreated mice served as control plasma. The inhibition of uptake of [^125^I]MIBG by human platelets was determined as described above.

### (Radio) chemicals

All SSRIs (alaproclate-HCl, citalopram-HBr, fluvoxamine-maleate, fluoxetine-HCl, norfluoxetine-HCl, paroxetine-HCl, sertraline-HCl) and other monoamine transporter inhibitors (imipramine-HCl, desipramine-HCl, clomipramine-HCl, maprotiline-HCl) were obtained from Sigma-Aldrich (St. Louis, USA). [^125^I]MIBG (specific activity ≈ 0.65 TBq/mmol) was purchased from Chelatec (Saint-Herblain, France). [^3^H]Serotonin (5-hydroxytryptamine creatinine sulfate, specific activity ≈ 3.84 TBq/mmol) was purchased from PerkinElmer.

### Data analysis

Unless stated otherwise, results are expressed as mean ± SD (*n*). Results were statistically analyzed using the Student *t* test and were considered significant if *p* < 0.05. Kinetic transport parameters (*K*_m_ and *V*_max_) and half maximal inhibitory concentration values (IC_50_) were calculated using nonlinear regression analysis in GraphPad Prism software (version 8; San Diego, USA). GraphPad Prism software was used to create all figures.

## Results

### Uptake and inhibition in SERT- and NET-transfected HEK293 cells

**Fig. 2:**
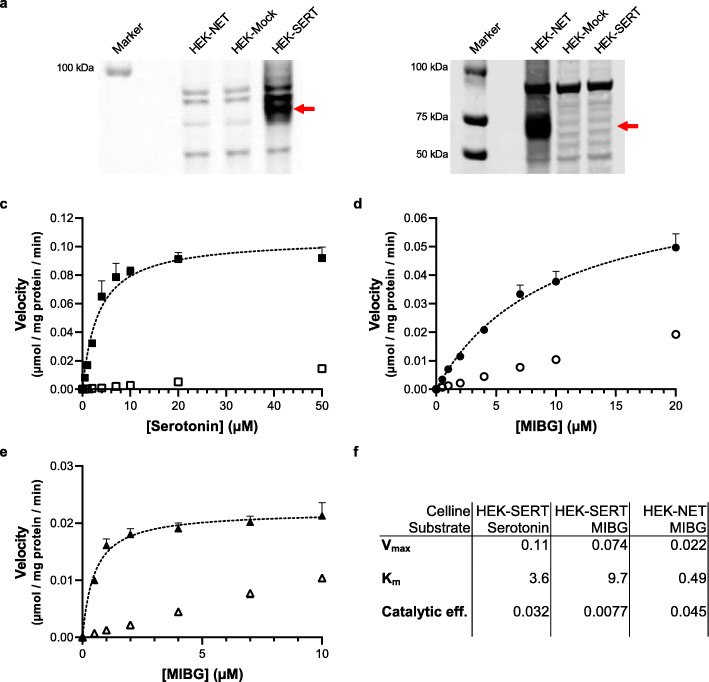
Uptake of [^3^H]serotonin and [^125^I]MIBG in SERT- and NET-transfected HEK293 cells. **a**, **b** Results of Western blotting analysis on samples of transfected HEK cells. **a** Primary antibody against human SERT. **b** Primary antibody against human NET. **c** Uptake of [^3^H]serotonin by HEK-SERT cells. **d** Uptake of [^125^I]MIBG by HEK-SERT cells. **e** Uptake of [^125^I]MIBG by HEK-NET cells. Closed symbols (■, ●, ▲) represent baseline-corrected, transporter-specific uptake. Open symbols (□, ○, Δ) represent non-specific cellular uptake observed in HEK-Mock cells. Cells were incubated with substrate for 1 h. The Michaelis-Menten graph is depicted as a black dashed line. **f** The kinetic parameters from this analysis: *V*_max_ in μmol/mg protein/min; *K*_m_ in μM; catalytic efficiency: ratio $\frac{V_{\mathrm{max}}}{K_m}$. Data represent mean ± SD (*n* = 2)

To determine the affinity of SERT for MIBG and its natural substrate serotonin, HEK293 cells were transfected with cDNA coding for SERT (HEK-SERT). In addition, HEK293 cells were transfected with cDNA coding for NET (HEK-NET) to investigate the affinity of NET for MIBG. The expression of recombinantly expressed SERT and NET was demonstrated by Western blotting analysis using primary antibodies against SERT (Fig. 2a) and NET (Fig. 2b). HEK-SERT cells rapidly accumulated [^3^H]serotonin (Fig. 2c). In contrast, no uptake of serotonin was observed for HEK293 cells transfected with the empty vector alone (HEK-Mock). Uptake assays using various substrate concentrations of [^3^H]serotonin confirmed saturability of substrate transport, with a high-affinity interaction (*K*_m_ = 3.6 μM). For the substrate [^125^I]MIBG, SERT performed as a low-affinity transporter (*K*_m_ = 9.7 μM; Fig. 2d) in comparison with NET (HEK-NET, *K*_m_ = 0.49 μM, Fig. 2e). The nonspecific uptake of serotonin represented only 3% of total uptake in SERT-transfected cells, whereas it accounted for 22% and 33% of MIBG uptake in SERT and NET transfected cells, respectively, at a substrate concentration of 10^−5^ M.

**Fig. 3:**
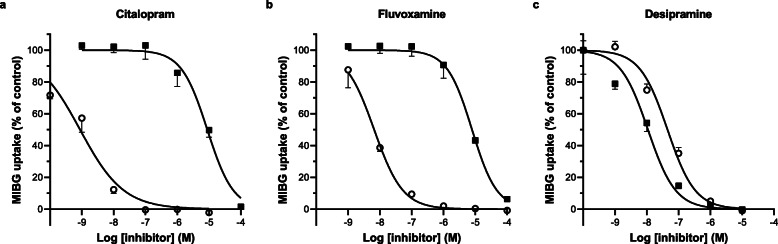
Inhibition of [^125^I]MIBG uptake in SERT- and NET-transfected HEK293 cells by SSRIs and desipramine. MIBG uptake assays were performed on HEK-SERT cells (open symbols ○) or HEK-NET cells (closed symbols ■) in the presence of various concentrations of citalopram (**a**), fluvoxamine (**b**), and desipramine (**c**). [^125^I]MIBG uptake is expressed relative to assays conducted in the absence of inhibitors. Cells were incubated for 1 h with a substrate concentration of 10^−6^ M. Data represent mean ± SD (*n* = 2)

To investigate the inhibitory potency of monoamine transporter inhibitors on the [^125^I]MIBG uptake by SERT and NET, the effect of the SSRIs citalopram and fluvoxamine was compared to the tricyclic antidepressant desipramine, an inhibitor with a selectivity for NET (see also Fig. 1). Citalopram (Fig. 3a) and fluvoxamine (Fig. 3b) both potently inhibited the uptake of [^125^I]MIBG in HEK-SERT cells with no significant inhibitory effect at low concentrations on HEK-NET cells. Desipramine inhibited the uptake of [^125^I]MIBG in both HEK-NET and HEK-SERT cells, with a stronger inhibitory effect in HEK-NET (Fig. 3c).

### In vitro selectivity of SSRIs in platelets and neuroblastoma cells

**Fig. 4:**
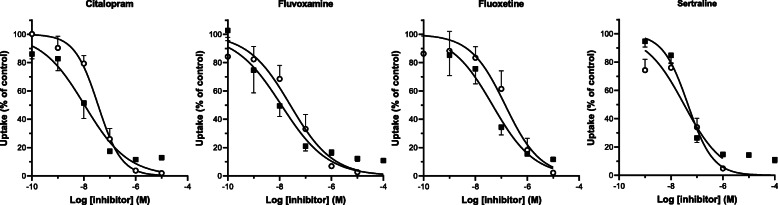
Inhibition of [^3^H]serotonin and [^125^I]MIBG uptake by SSRIs in human platelets. Inhibition of [^3^H]serotonin uptake is depicted by open symbols (○), and inhibition of [^125^I]MIBG uptake by closed symbols (■). Samples were incubated at 37 °C for 15 min (serotonin) or 4 h (MIBG) with a substrate concentration of 10^−8^ M. Substrate uptake is expressed as the percentage of control without inhibitor. Data represent mean ± SD (*n* = 4–7)

Next, we tested whether the SSRIs can effectively block the uptake of [^3^H]serotonin and [^125^I]MIBG in human platelets. Co-incubation with the SSRIs citalopram, fluvoxamine, fluoxetine, and sertraline showed effective, inhibitor concentration-dependent inhibition of the uptake of both substrates (Fig. 4). The degree of inhibition by these SSRIs was very similar for both serotonin and MIBG, indicating that both substrates are taken up by the same transporter (SERT). The total uptake (percentage of added radioactivity) in the control samples without SSRIs amounted to 57.5 ± 13.8% for serotonin (*n* = 5) and 23.6 ± 3.6% for MIBG (*n* = 17). Nonspecific uptake (percentage of controls) was 2.6 ± 0.4% for serotonin and 12.3 ± 2.5% for MIBG.

**Fig. 5:**
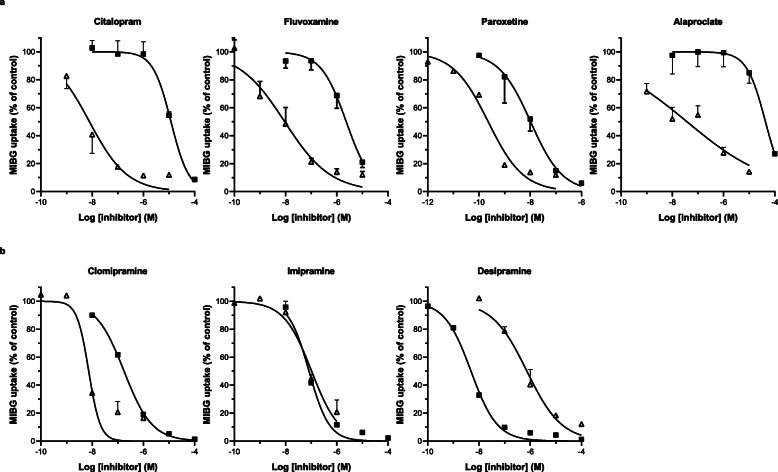
Inhibition of [^125^I]MIBG uptake by SSRIs and tricyclic antidepressants in human platelets and cultured SK-N-SH neuroblastoma cells. [^125^I]MIBG uptake in isolated human platelets is depicted by open symbols (Δ), and [^125^I]MIBG uptake in cultured human SK-N-SH neuroblastoma cells by closed symbols (■). Samples were incubated at 37 °C for 4 h with a substrate concentration of 10^−8^ M. MIBG uptake is expressed as the percentage of control without inhibitor. Data represent mean ± SD (*n* ≥ 3)

In the subsequent experiments, the effect of different monoamine transporter inhibitors on the [^125^I]MIBG uptake was compared between human platelets (uptake via SERT) and human SK-N-SH neuroblastoma cells (uptake via NET). The tested SSRIs (Fig. 5a) were more potent in inhibiting the MIBG uptake in platelets than in SK-N-SH cells. For example, citalopram at a concentration of 10^−7^ M nearly fully inhibited the uptake of MIBG in platelets with negligible effect on the MIBG uptake in SK-N-SH neuroblastoma cells. To compare the effects of the SSRIs with monoamine transporter inhibitors from the tricyclic antidepressant family, we included clomipramine, imipramine, and desipramine in our analysis (Fig. 5b). Imipramine and desipramine, while able to inhibit the MIBG uptake in platelets, also have a profound inhibitory effect on the uptake of MIBG in neuroblastoma cells. This difference in selectivity is in line with earlier described selectivity factors (i.e., clomipramine: more SERT selective; imipramine: NET ≈ SERT; desipramine: more NET selective; see also Fig. 1) [24].

The potency and selectivity of all SSRIs tested in comparison with the tricyclic antidepressants and maprotiline, an inhibitor with high selectivity for NET, are listed in Table 1 [4, 25]. The most potent inhibitor of the [^125^I]MIBG uptake in platelets was paroxetine with an IC_50_ of 0.2 nM. We defined the selectivity factor as the IC_50_ ratio of the MIBG uptake in platelets over that in SK-N-SH cells. Of the seven tested SSRIs, citalopram proved to be the most selective MIBG inhibitor, being approximately 1500 times more potent in inhibiting the MIBG uptake in platelets than in SK-N-SH neuroblastoma cells. Comparable results were obtained with platelets from healthy volunteers (data not shown).

**Table 1:** The potency and selectivity of all tested monoamine transporter inhibitors

		IC_50_ (nM) of uptake inhibition			Selectivity factor
		Platelets		SK-N-SH	IC_**50**_ ratio
		[^3^H]Serotonin	[^125^I]MIBG	[^125^I]MIBG	
SSRIs	Alaproclate	516	38	43420	**1158**
	Citalopram	32	7.8	11940	**1521**
	Fluvoxamine	30	9.3	2339	**252**
	Fluoxetine	115	32	1270	**40**
	Norfluoxetine	107	24	2760	**115**
	Paroxetine	0.6	0.2	10	**45**
	Sertraline	41	36	3301	**91**
TCAs	Clomipramine	18	7	176	**24**
	Imipramine	118	104	82	**0.8**
	Desipramine	648	744	5	**0.007**
TeCA	Maprotiline	n.d.	1000–10,000	39	**0.04–0.004**

Selective inhibition of 10^−8^ M [^125^I]MIBG or [^3^H]serotonin uptake by monoamine transporter inhibitors in human platelets or SK-N-SH neuroblastoma cells in vitro. Selectivity was expressed as the IC_50_ ratio of the MIBG uptake in SK-N-SH cells over that in platelets. *SSRI* Selective serotonin reuptake inhibitor; *TCA* Tricyclic antidepressant; *TeCA* Tetracyclic antidepressant

MIBG uptake and inhibition studies with PC12 pheochromocytoma cells were included to investigate the role of cytoplasmic storage granules. The IC_50_ values of the monoamine transporter inhibitors for the inhibition of MIBG uptake in PC12 cells proved to be similar to those observed in the assays with the SK-N-SH neuroblastoma cells (data not shown), indicating that the MIBG uptake inhibitors only interfered with transport across the outer cell membrane and not with granular accumulation.

### Effect of monoamine transporter inhibitors on the [^125^I]MIBG tumor uptake in vivo

**Fig. 6:**
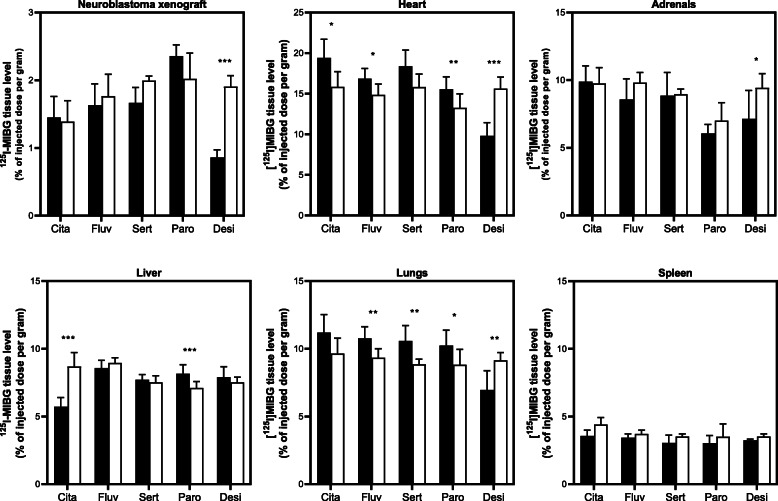
The in vivo effect of SSRIs and desipramine on [^125^I]MIBG levels in neuroblastoma xenografts and normal tissues. Before [^125^I]MIBG injection, mice received either an inhibitor (black bars) or sodium chloride (white bars; control). From left to right, the results of citalopram (20 mg/kg), fluvoxamine (50 mg/kg), sertraline (20 mg/kg), paroxetine (2 mg/kg), and desipramine (20 mg/kg) are shown. Data represent mean + SD with *n* = 3–6 for fluvoxamine, *n* = 4 for sertraline, *n* = 4–7 for desipramine, and *n* = 7–9 for citalopram. Please note the different scales of the *Y*-axis. Asterisks were used to indicate significance levels: *p < 0.05, **p ≤ 0.01, and ***p ≤ 0.001

Next, we studied in an in vivo model of SK-N-SH neuroblastoma-xenografted mice whether plasma drug levels, that prevent platelet MIBG uptake, could be realized without affecting radioiodinated MIBG tumor uptake. The administered doses of desipramine (20 mg/kg) and the four in vitro most potent SSRIs citalopram (20 mg/kg), fluvoxamine (50 mg/kg), sertraline (20 mg/kg), and paroxetine (2 mg/kg) were well tolerated, as neither detectable motor nor behavioral abnormalities were observed. The [^125^I]MIBG accumulation in the neuroblastoma xenografts (Fig. 6) was not significantly affected by treatment with any of the four SSRIs, but was diminished by 60% in mice treated with desipramine, as expected from our in vitro studies with desipramine and SK-N-SH cells.

Similar in vivo studies with mice bearing PC12 pheochromocytoma xenografts resulted in no significant difference in tumor MIBG uptake after treatment with citalopram and sertraline (data not shown). In contrast to our results obtained with neuroblastoma xenografts, fluvoxamine reduced the pheochromocytoma-associated radioactivity with 64% (*p* < 0.002). Paroxetine was not tested. Desipramine treatment of mice bearing PC12 pheochromocytoma xenografts resulted in the same reduction of MIBG uptake as seen in SK-N-SH neuroblastoma xenografts (*p* < 0.004).

Regarding normal tissues (Fig. 6), SSRI treatments had no significant effect on the [^125^I]MIBG levels in the blood, intestines, ears/skin, kidneys, brains (data not shown), spleen, and adrenals. For the liver, MIBG levels after SSRI treatment were highly variable and contradictory. The [^125^I]MIBG concentration in the livers of mice undergoing fluvoxamine or sertraline treatment was not significantly different from controls. Citalopram treatment, however, led to a 35% decrease in MIBG levels, whereas paroxetine resulted in a 17% increase. In addition, we observed consistent higher (14–25%) [^125^I]MIBG levels in the heart and lungs in the SSRI-treated mice. Desipramine significantly reduced the [^125^I]MIBG uptake of the heart, lungs, and adrenals with 24–37%, but this reduction was less pronounced than in neuroblastoma xenografts, as described above.

### Ex vivo bioassay of murine plasma SSRI activity

**Fig. 7:**
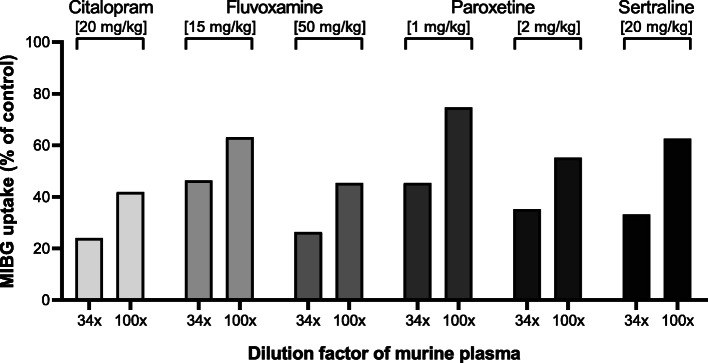
Inhibition of [^125^I]MIBG uptake in isolated human platelets by different dilutions of murine plasma obtained from SSRI-treated mice. One hour before collecting plasma, mice intraperitoneally received the monoamine transporter inhibitor at the dose indicated in parenthesis. Data are from a representative experiment performed in duplicate and were confirmed by at least 2 experiments performed at slightly different conditions

To assess the inhibitory potency of plasma of mice treated with the different SSRIs as described above, this plasma was added to human platelets to measure the inhibition of [^125^I]MIBG uptake in platelets. The results depicted in Fig. 7 demonstrate that the SSRI drug levels in the murine circulation highly exceeded those required for effective inhibition of [^125^I]MIBG uptake in human platelets.

## Discussion

The most common side effect encountered after [^131^I]MIBG therapy is hematological toxicity, in particular thrombocytopenia. Since platelets are anucleate cells with a finite lifespan, and the observed thrombocytopenia is not an instantaneous toxicity, it is conceivable that platelet precursor cells, i.e., megakaryocytes located in the bone marrow, are the primary targets of radiotoxic MIBG. The uptake of MIBG by HEK-SERT cells and in platelets as observed in our study is in line with a previous study [18], demonstrating the promiscuity of MIBG for two related but different monoamine transporters, namely SERT and NET. Moreover, we showed that SSRIs were effective in reducing the MIBG uptake in human platelets. Since both platelets and their precursor cells, megakaryocytes, express the serotonin transporter on their cell membrane [26, 27], the application of SSRIs could be used to circumvent [^131^I]MIBG-induced thrombocytopenia.

We have, therefore, investigated whether inhibition of MIBG accumulation in platelets by SSRIs is feasible without affecting MIBG tumor uptake in neuroblastoma, using in vitro and in vivo models. Our finding that paroxetine is both the most potent serotonin and MIBG inhibiting SSRI is consistent with previous investigations [24, 25, 28–31]. That potency and selectivity do not coincide can be appreciated by comparing paroxetine and citalopram. In our in vitro MIBG uptake assays, the most selective drug proved to be citalopram, being 1500-fold more potent in preventing uptake of MIBG in platelets than in SK-N-SH neuroblastoma cells. These results are consistent with previous studies, which showed that citalopram is the most selective SSRI, being a far more potent inhibitor of SERT than of NET [24, 25, 32, 33].

In our in vivo model of mice bearing a human neuroblastoma xenograft, none of the tested SSRIs significantly affected MIBG accumulation in the tumor. Intervention with any of the four tested SSRIs led to a slight increase of MIBG levels in the heart and lungs. It is known that the instant effect of short-term SSRI treatment is suppression of sympathetic nervous system activity [34, 35]. Presumably, this suppression results in more “leftover capacity” for NET to transport MIBG intracellular in the sympathetic end organs. In contrast, a recent in vivo rabbit study found no impact of the SSRI escitalopram on cardiac [^123^I]MIBG uptake [36]. In the liver, citalopram and paroxetine had diametrically opposed effects on the MIBG accumulation. The serotonin transporter, localized on hepatocytes and on hepatic stellate cells, plays an important, although not completely understood, role in hepatic repair after injury [37, 38]. One would expect, therefore, that administration of a SSRI would be accompanied by a decrease in hepatic MIBG uptake, such as we encountered with citalopram. Administration of paroxetine, however, led to a small increase in hepatic MIBG uptake. At this moment, the underlying mechanism that could account for the observed results remains to be elucidated.

The intended purpose of a pharmacological intervention with a SSRI would be to prevent thrombocytopenia due to [^131^I]MIBG uptake after the infusion of a therapeutic dose of radiotoxic MIBG. To accomplish this, our desired SSRI should be effective for a discrete period of time after a single ingestion. The primary metabolite of citalopram, desmethylcitalopram, is only slightly less potent and selective than citalopram in inhibiting SERT in vitro [24, 39]. After oral ingestion, citalopram is completely absorbed: maximum plasma drug levels are reached within 4 h, and plasma half-life of 33–54 h is appropriate for the objective stated above [40, 41]. Fluvoxamine also has favorable pharmacokinetic properties [42]. However, a case report of an 11-year-old boy whose ingestion of a single dose of fluvoxamine resulted in serotonin syndrome [43] led us to conclude that citalopram seems the most suitable candidate to protect the platelets and precursor megakaryocytes from intracellular radiation in patients undergoing MIBG therapy. Citalopram has some additional advantages over the other SSRIs. For instance, fluoxetine (1–4 days) and its primary and active metabolite, norfluoxetine (7–15 days), have very long elimination half-lives [44]. Sertraline is not applicable for single-dose administration because only 44% is absorbed after oral ingestion [45]. Paroxetine undergoes extensive first-pass metabolism, reducing its bioavailability to approximately 50% [46].

Citalopram is capable of fully and selectively reducing MIBG uptake in platelets, without interfering with MIBG tumor uptake. Moreover, plasma concentrations of citalopram that will inhibit SERT [47] had no effect on platelet function [48], making it highly unlikely that a single ingestion of citalopram will have an effect on hemostasis and thrombosis [49]. Combined with the above-described favorable pharmacokinetic properties, we recommend citalopram for further preclinical and clinical studies on the prevention of MIBG accumulation in megakaryocytes. Previous studies failed to demonstrate MIBG uptake in megakaryocytic cell lines [50] and in human megakaryocytes [51], probably due to suboptimal in vitro culture conditions. A case report of osteomedullary MIBG uptake in a 13-month-old patient with acute megakaryocytic leukemia argues, however, in favor of the capacity of cells from the megakaryocytic lineage to concentrate MIBG [52]. Recently, SERT expression in human megakaryoblastic MEG-01 cells has been demonstrated unequivocally by different research groups [26, 27]. In ongoing studies, with optimized experimental conditions, we will investigate the inhibitory potential of SSRIs on the MIBG accumulation in megakaryocytes, bone marrow supporting stroma cells, and other hematopoietic cells, to further elucidate the underlying mechanisms of MIBG-related toxicity.

## Conclusions

Selective serotonin reuptake inhibitors (SSRIs) are able to prevent MIBG uptake in platelets without affecting neuroblastoma tumor uptake in an in vivo mouse model. The concomitant application of a SSRI during [^131^I]MIBG therapy appears to be a promising strategy to prevent thrombocytopenia in neuroblastoma patients. Future studies will investigate the inhibitory potential of SSRIs on the MIBG accumulation in the parent cells responsible for the production of platelets, human megakaryocytes.

## Supplementary information

**Additional file 1: Supplementary Table I.** A summary of reuptake inhibitor studies in rodents using behavioral or biochemical analysis to determine the effective dose

## Data Availability

The datasets generated during and/or analyzed during the current study are available from the corresponding author on reasonable request.

## Abbreviations

SSRI: Selective serotonin reuptake inhibitor
MIBG: Meta-iodobenzylguanidine
NET: Norepinephrine transporter
SERT: Serotonin transporter
IC_50_: Half maximal inhibitory concentration
ATCC: American Type Culture Collection
PRP: Platelet-rich plasma
HBSS: Hank’s Balanced Salt Solution
s.c.: Subcutaneous
IP: Intraperitoneal
HEK: Human embryonic kidney
