# Supplementary material for: Selective serotonin reuptake inhibitors (SSRIs) prevent meta-iodobenzylguanidine (MIBG) uptake in platelets without affecting neuroblastoma tumor uptake
Source: EJNMMI Res. 2020 Jul 8;10:78. doi: 10.1186/s13550-020-00662-w (PMC7343696; doi:10.1186/s13550-020-00662-w)
Supplement: Supplementary file 1 — Additional file 1: Supplementary Table I. A summary of reuptake inhibitor studies in rodents using behavioral or biochemical analysis to determine the effective dose [file 13550_2020_662_MOESM1_ESM.docx]

**Supplementary table I.** A summary of reuptake inhibitor studies in rodents using behavioral or biochemical analysis to determine the effective dose

| Drug^a^ | Reported treatments | | | Type of assay | Reference |
| --- | --- | --- | --- | --- | --- |
|  | species | dose^c^  (mg/kg) | route |  |  |
| Citalopram | mice | 0.9^c^ | i.p. | I | (1) |
| (20 mg/kg) | mice | 1.2^c^ | oral | I | (2) |
|  | mice | 24 | i.v. | II | (3) |
|  | rats | 6^c^ | oral | III | (2, 4) |
|  |  |  |  |  |  |
| Paroxetine | mice | 0.2^c^ | i.p. | I | (1) |
| (2 mg/kg) | mice | 1 - 10^c^ | i.p. | III | (5) |
|  | rats | 1.9^c^ | oral | III | (4) |
|  |  |  |  |  |  |
| Sertraline | mice | 1.5^c^ | i.p. | I | (1) |
| (20 mg/kg) | mice | 1.0^c^ | oral | I | (2) |
|  | rats | 2.8^c^ | i.p. | III | (6) |
|  |  |  |  |  |  |
| Fluvoxamine | mice | 6^c^; 35^c^ | i.p. | I | (1, 7) |
| (50 mg/kg) | mice | 13^c^ | oral | I | (2) |
|  | rats | 25 | i.p. | III | (7) |
|  |  |  |  |  |  |
| Desipramine | mice | >39^c^ | i.p. | I | (1) |
| (20 mg/kg) | mice | 20 – 320^c^ | oral | I | (2, 4, 7) |
|  | mice | 5 – 36 | i.p. | IV | (8) |
|  | rats | >30 | oral | III | (2) |

1. The mg/kg dose applied in this study is given in parenthesis
2. Reports deal with different analysis as listed below:
3. Potentiation of tryptophan or 5-hydroxytryptophan induced symptoms
4. Plasma level was 6 mM, fifteen min after administration,
5. Inhibition of 5HT uptake in (fore) brain or hypothalamic synaptosomes (ex vivo)
6. Antagonism of amphetamine-stimulated locomotor activity
7. 50 % effective doses

**References**

1. Hyttel J. Pharmacological characterization of selective serotonin reuptake inhibitors (SSRIs). Int Clin Psychopharmacol. 1994;9 Suppl 1:19-26.

2. Feighner JPB, W.F. . Selective serotonin re-uptake inhibitors; Advances in basic research and clinical practice. Feighner JPB, W.F. , editor. Chichester, England: John Wiley and Sons; 1996.

3. Fredricson Overo K. Kinetics of citalopram in test animals; drug exposure in safety studies. Prog Neuropsychopharmacol Biol Psychiatry. 1982;6(3):297-309.

4. Thomas DR, Nelson DR, Johnson AM. Biochemical effects of the antidepressant paroxetine, a specific 5-hydroxytryptamine uptake inhibitor. Psychopharmacology (Berl). 1987;93(2):193-200.

5. Magnussen I, Tønder K, Engbaek F. Paroxetine, a potent selective long-acting inhibitor of synaptosomal 5-HT uptake in mice. Journal of Neural Transmission. 1982;55(3):217-26.

6. Koe BK. Preclinical pharmacology of sertraline: a potent and specific inhibitor of serotonin reuptake. The Journal of clinical psychiatry. 1990.

7. Claassen V, Davies J, Hertting G, Placheta P. Fluvoxamine, a specific 5‐hydroxytryptamine uptake inhibitor. British journal of pharmacology. 1977;60(4):505-16.

8. Tyler TD, Tessel RE. Norepinephrine uptake inhibitors as biochemically and behaviorally selective antagonists of the locomotor stimulation induced by indirectly acting sympathomimetic amines in mice. Psychopharmacology. 1980;69(1):27-34.
